# Factors influencing agreement between child self-report and parent proxy-reports on the Pediatric Quality of Life Inventory™ 4.0 (PedsQL™) generic core scales

**DOI:** 10.1186/1477-7525-4-58

**Published:** 2006-08-30

**Authors:** Joanne Cremeens, Christine Eiser, Mark Blades

**Affiliations:** 1 Division of Behavioral Medicine, St. Jude Children's Research Hospital, Memphis, TN, USA; 2 Department of Psychology, University of Sheffield, UK

## Abstract

**Background:**

In situations where children are unable or unwilling to respond for themselves, measurement of quality of life (QOL) is often obtained by parent proxy-report. However the relationship between child self and parent proxy-reports has been shown to be poor in some circumstances. Additionally the most appropriate statistical method for comparing ratings between child and parent proxy-reports has not been clearly established. The objectives of this study were to assess the: 1) agreement between child and parent proxy-reports on an established child QOL measure (the PedsQL™) using two different statistical methods; 2) effect of chronological age and domain type on agreement between children's and parents' reports on the PedsQL™; 3) relationship between parents' own well-being and their ratings of their child's QOL.

**Methods:**

One hundred and forty-nine healthy children (5.5 – 6.5, 6.5 – 7.5, and 7.5 – 8.5 years) completed the PedsQL™. One hundred and three of their parents completed these measures in relation to their child, and a measure of their own QOL (SF-36).

**Results:**

Consistency between child and parent proxy-reports on the PedsQL™ was low, with Intra-Class correlation coefficients ranging from 0.02 to 0.23. Correlations were higher for the oldest age group for Total Score and Psychosocial Health domains, and for the Physical Health domain in the youngest age group. Statistically significant median differences were found between child and parent-reports on all subscales of the PedsQL™. The largest median differences were found for the two older age groups. Statistically significant correlations were found between parents' own QOL and their proxy-reports of child QOL across the total sample and within the middle age group.

**Conclusion:**

Intra-Class correlation coefficients and median difference testing can provide different information on the relationship between parent proxy-reports and child self-reports. Our findings suggest that differences in the levels of parent-child agreement previously reported may be an artefact of the statistical method used. In addition, levels of agreement can be affected by child age, domains investigated, and parents' own QOL. Further studies are needed to establish the optimal predictors of levels of parent-child agreement.

## Background

A number of well-validated quality of life (QOL) measures for adults have been developed, many of which are used in routine clinical trials. The inclusion of QOL measures in clinical trials has in part come from increasing recognition that self-reports on subjective states can provide information about the consequences of treatment plans (such as behavioral or psychological outcomes) that may not be captured by traditional outcome indices [1]. In the last twenty five years, a number of well-validated child instruments have been developed [2].

Given the lower cognitive and language skills of young children, the majority of child QOL instruments have been developed for children above eight years with proxy reports (usually parent) used to gain information about younger children [3]. However the value of obtaining children's self-reports about their health, functioning, abilities, and emotions is increasingly recognized within both medical care and child health research [2]. Several generic and disease-specific QOL measures are now available that include parallel child and parent proxy-report versions (for example, generic: the Pediatric Quality of Life Inventory™ (PedsQL™) [4,5], the Child Health and Illness Profile – Child Edition (CHIP-CE™) [6], and the KINDL™ [7], disease-specific: the Cystic Fibrosis Questionnaire (CFQ) [8], the Child Health Ratings Inventory (CHRIs) [9], and the How Are You? (HAY) [10]).

The availability of measures with parallel child and parent versions has raised questions about the level of agreement between children's own views and those of their parents about child functioning. The literature is relatively confused, with reported of poor parent-child agreement [e.g., [11,12]], and of moderate to high agreement [e.g., [13,14]]. Parent-child agreement may be affected by a number of variables [15]. In a review of the relationship between child and parent QOL ratings, Eiser and Morse [16] concluded agreement is dependent on the domain being measured, with higher agreement for physical aspects of health compared to emotional or social aspects. Eiser and Morse [16] also reported evidence of higher agreement between parents and *chronically sick *children compared with parents and *healthy *children. Some researchers have found evidence that parents of sick children tend to underestimate their child's QOL compared with children's own ratings [e.g., [17]]. The reverse (i.e., overestimation) has been reported with parents of healthy children [e.g., [13,18,19]].

Agreement between child and parent proxy-ratings may also vary by the age of the child. Eiser and Morse [16] identified only two studies examining the effect of age [4,13]. Varni et al. [4] reported that agreement was highest between *children *with cancer and their parents for cognitive functioning, and highest between *adolescents *and parents for physical functioning. Theunissen, Vogels, Koopman, Verrips, Zwinderman, and Verloove-Vanhorick [13] found that parent-child agreement was related to child's age and their positive emotions ratings. Specifically, Theunissen et al. [13] reported that older children (10–11 years) with low positive emotion scores agreed less with their parents than younger children (8–9 years), and older children with high positive emotion scores agreed more with their parents. A study by Annett, Bender, DuHamel, and Lapidus [20] with children with asthma reported parent-child agreement increased with child age. Ronen, Streiner, and Rosenbaum [21] reached similar conclusions, with younger age predicting greater differences between parents and children with epilepsy.

An additional factor for consideration here is the impact of parents' own functioning and well-being. Eiser, Eiser, and Stride [22] found that mothers who rated their own well-being as poor also rated their child's QOL as poor, suggesting that parents project their own feelings on to judgments about the child's functioning. In addition, Goldbeck and Melches [23] reported a significant interaction effect of parental QOL and patients' self-reported QOL in predicting parental proxy reports of their children's QOL.

Part of the confusion described above may relate to the statistical methods employed to compute parent-child agreement. The most frequently used statistic for examining agreement between child and parent reports has been the Pearson product-moment correlation coefficient [16]. However Pearson *r *values provide information on the covariation among scores but do not indicate absolute agreement [24]. A more appropriate statistic for examining agreement between raters is the Intra-class correlation coefficient (ICC). ICC values provide an index that reflects the ratio between subject variability and total variability [25].

It is useful to examine mean differences between children's and parents' reports, as it is possible for their scores to be correlated (i.e., linearly related) but also show statistically significant differences in mean scores [26]. Analyses which include both correlation and mean difference testing are needed in order to provide more conclusive evidence regarding the relationship between parent and child ratings. We identified two studies which adopted this approach [26,27]. Both assessed parent-child agreement for QOL ratings in pediatric cancer populations. These researchers found moderate correlations between child and parent scores and no group differences between their mean scores [26,27]. It is questionable if test scores displaying moderate correlations can be considered equivalent. Correlation coefficients of at least 0.70 are usually required for a reasonable prediction of individual test scores [28].

Our goal was to extend knowledge of the factors influencing child-parent agreement in rating child QOL in healthy populations. First, we considered differences in agreement across two different statistical methods. Intra-Class correlation coefficients (ICC) were used to evaluate correlational consistency between child and parent scores on the generic core scales of the UK-English version of the Pediatric Quality of Life Inventory™ 4.0 (PedsQL™) [4,5], and Wilcoxon median testing to evaluate agreement between child and parent ratings on this measure. Second, we considered the effect of chronological age and domain type (i.e., physical vs. psychosocial aspects) on agreement between children's and parents' reports on the PedsQL™. We assessed parent-child agreement across three age groups (stratified by year in school), and across the Physical Health domain and Psychosocial Health domains. Third, we investigated the relationship between parents' own well-being and their ratings of their child's QOL.

Based on the findings of Eiser and Morse [16], we predicted that in this healthy sample parent-child correlations would be low to moderate. Furthermore, we expected statistically significant group differences in child and parent median scores, specifically parents' scores would be higher than children's scores due to the over-estimation effects found in previous studies with healthy child populations [13,18,19]. In relation to chronological age, we expected that parent-child agreement would increase with child age, based on the findings of previous work [4,20,21]. In relation to domain type, we expected that parent-child correlations would be higher for the physical health compared to psychosocial health domains. Finally following on from the findings of Eiser, Eiser and Stride [22] on the effect of mother's own well-being on their ratings of their children's QOL, we expected that parents' own QOL levels would be correlated to their proxy-reports of child QOL.

## Methods

### Sample

Participants were 149 English-speaking healthy children (67 girls, 82 boys) between the ages of 5.5 and 8.5 years (*M *= 7.33, *SD *= 0.85) recruited from three UK schools, in the south-east of England. Children were excluded if they were receiving any treatment for a chronic or acute medical condition, or if they had a history of special needs or learning difficulties. Children were stratified into three age groups based on UK school year group (5.5–6.5 years, *n *= 41, *M *age = 6.20; 6.5–7.5 years, *n *= 53, *M *age = 7.29; 7.5–8.5 years, *n *= 55, *M *age = 8.22). Ninety-seven percent were Caucasian, 3% were of Asian origin.

One hundred and three of their parents returned the questionnaires for proxy-report, yielding a response rate of 69%. Therefore, 103 parent-child dyads were used in this study (5.5–6.5 years, *n *= 29, 6.5–7.5 years, *n *= 34, 7.5–8.5 years, *n *= 40). There were no statistically significant differences in race or gender between the children whose parents returned the questionnaires (n = 103) and children whose parents did not return the questionnaires (n = 46). Ethics approval was given by the Department of Psychology Ethics Committee, University of Sheffield. Written consent from parents and verbal assent from children were obtained.

### Procedure

Children were interviewed individually in a quiet room separate from their classroom. Children were given the UK-English version of the PedsQL™ 4.0 generic core module, administered as directed by the PedsQL™ manual [28-30]. Parents completed the UK-English version of the PedsQL™ 4.0 generic core module in relation to their child, and the SF-36 scale in relation to themselves. These questionnaires were sent home with each child for parents to complete (*n *= 103 returned, yielding a high return rate of 69%).

### Measures

#### Self and parent proxy-reported child QOL

##### PedsQL^™^4.0 measure

The PedsQL™ generic core module includes parallel child self-report and parent proxy-report versions for ages 5–18 years, differing only in wording and length of response scale. In this study, the young child self-report version of PedsQL™ was used. The young child self-report version employs a 3-point Likert scale going from 'not at all' to 'a lot' with smiley faces to aid in the rating task. Items on parent version are virtually identical to the child version, with minor language changes. The parallel parent version uses a 5-point Likert response scale going from 'never' to 'almost always'.

The generic core scale comprises 23 items that contribute to a Total Score and four subscales: physical functioning, emotional functioning, social functioning and school functioning. It has been shown that scores on the subscale Physical Functioning can be used to produce a single Physical Health Summary Scale, while the remaining subscales can be used as a single Psychosocial Health Summary Scale [5]. The PedsQL™ was developed in the U.S., and the reliability and validity is well-established [5,29-31]. This measure has been widely used in research and translated into many languages. Measurement properties for the UK-English version are equivalent to the original PedsQL™ developed in American-English [32].

##### Parent QOL

The SF-36 scale [33] was included as a measure of parents' own well-being. This measure includes eight subscales, with varying number of items and response formats, defined as physical functioning, role limitation (physical), role limitation (social), social functioning, mental health, energy/vitality, pain and general health perception. This measure has established psychometric properties, and has been used extensively in research [34].

### Treatment of results and statistical analyses

The PedsQL™ 4.0 measure was scored as described in original publications and manuals. Children's and parents' responses to all items were reverse scored and linearly transformed to a 0–100 scale (i.e., 0 = 100, 1 = 75, 2 = 50, 3 = 25, 4 = 0), with higher scores indicating higher QOL [5]. Total Score, Physical Health and Psychosocial Health scores were used in the analyses. For the SF-36 measure, we calculated a Total Score as described by Eiser, Eiser and Stride [22] with higher scores indicating higher QOL.

The internal reliability (Cronbach's alpha coefficients) for PedsQL™ 4.0 was calculated. We assumed a minimum standard of 0.70 for Cronbach's alpha coefficients for adequate internal reliability [35]. Range of measurement for the PedsQL™ was determined based on the percentage of scores at the extreme of the scaling range. Kolmogorov-Smirnov tests were used to assess whether the distributions of children's and parent's PedsQL™ scores were normally distributed. Where data was significantly skewed or different from a normal distribution, non-parametric statistics were used in the analyses [36].

Agreement between child self and parent proxy-report on the PedsQL™ 4.0 was assessed using ICC values and median difference testing using Wilcoxon significance tests. This analysis was conducted for the total sample and separately for the three age groups. The relationship between parent QOL (SF-36 scores) and parent-rated child QOL (PedsQL™ scores) was assessed using Spearman's correlation coefficients, for the total sample and separately for the three age groups.

## Results

### Internal reliability

Cronbach's alpha coefficients for child self and parent proxy-report Total Scores and subscales scores on the PedsQL™ all exceeded the 0.70 standard, with the exception of Physical Health for child self-report (0.46, Table 1).

**Table 1:** Child-rated and parent-rated child QOL on the PedsQL™ measure: means, reliabilities and scale statistics

**Scale**	**N**	**Mean (SD)**	**No. of items**	**Alpha (α)**	**Percentage floor**	**Percentage ceiling**
**PedsQL™**						
**Child Self-report**						
Total Score	149	71.77 (14.40)	23	0.81	0	0
Physical Health	149	76.42 (14.01)	8	0.46	5.4	0
Psychosocial Health	149	68.90 (16.24)	5	0.76	1.3	0
**Parent Proxy-report**						
Total Score	149	79.97 (11.73)	23	0.91	0.7	0
Physical Health	149	86.10 (11.41)	8	0.73	10.7	0
Psychosocial Health	149	76.72 (13.00)	15	0.89	0.7	0

### Range of measurement

Table 1 presents means and percentage of scores at the floor and ceiling for self and proxy-report. No ceiling effects were found for self or proxy-report PedsQL™ Total Scores and subscale scores. However, minimal floor effects existed for both self and proxy-report (ranged from 0.7% to 10.7%, Table 1). Both self and proxy-reported PedsQL™ scores were significantly skewed towards the higher end of the scale (Table 1).

### Consistency and agreement between self and proxy-reported child QOL

#### Correlational consistency

Intra-class correlation coefficients between child self-report and parent proxy-report on the PedsQL™ are presented in Table 2. The level of agreement between self and proxy-reports was low. Correlations were higher for Total Score and Psychosocial Health for the oldest age group (0.23 and 0.22 respectively) than for the other age groups. For the youngest age group correlations were higher for Physical Health (0.21) than for the other age groups.

**Table 2:** Correlations between child self and parent proxy-rated child QOL

	**Intra-class correlation coefficient (ρ_I_)**			
**Scale**	**Total sample (n = 103)**	**5.5 – 6.5 years (n = 29)**	**6.5 – 7.5 years (n = 34)**	**7.5 – 8.5 years (n = 40)**
**PedsQL™**				
Total Score	0.09	0.03	0.06	0.23*
Physical Health	0.02	0.21*	0.10	0.14
Psychosocial Health	0.12	0.08	0.06	0.22*

Denotes statistically significant child/parent correlation at *** p < .001, ** p < .01, or * p < .05.

#### Agreement in median scores

**Figure 1:**
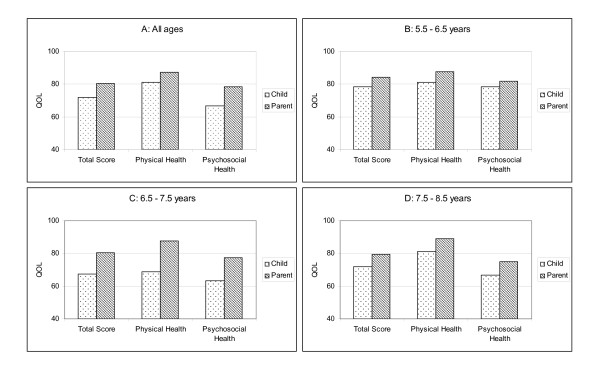
Median scores on the PedsQL™ for child self-report and parent proxy-report across age groups.

Agreement between self and proxy-report median scores on the PedsQL™ are presented in Table 3. Self and proxy-report PedsQL™ scores were statistically significantly different for Total Score, Physical Health and Psychosocial Health (all at *p *< .001 level, Table 3), because parents reported *better *child QOL than did their children. Psychosocial Health scores showed the largest median difference between self and proxy-report (median difference = 11.66). Self and proxy-report showed higher differences in the older age groups (6.5 – 7.5 years and 7.5 – 8.5 years) than the youngest age group (5.5 – 6.5 years, Table 3). The largest differences were found in the middle age group (6.5 – 7.5 years, differences ranged from 13.04 to 18.75). These median differences are displayed graphically in Figure 1.

**Table 3:** Median differences between child self and parent proxy-rated child QOL

	**Median (Mean)**			
**Scale**	**Total sample (n = 103)**	**5.5 – 6.5 years (n = 29)**	**6.5 – 7.5 years (n = 34)**	**7.5 – 8.5 years (n = 40)**
**PedsQL™**				
**Total Score**				
Child	71.74 (71.77)	78.26 (77.60)	67.39 (67.34)	71.74 (71.58)
Parent	80.43*** (79.97)	84.24 (80.75)	80.43*** (80.34)	79.35** (70.10)
**Physical Health**				
Child	81.24 (76.42)	81.25 (79.88)	68.75 (71.81)	81.25 (78.18)
Parent	87.43*** (86.10)	87.50 (84.59)	87.50*** (87.41)	89.06** (86.09)
**Psychosocial Health**				
Child	66.67 (68.90)	78.33 (76.57)	63.33 (64.44)	66.67 (67.52)
Parent	78.33*** (76.72)	81.67 (78.69)	77.50*** (76.57)	75.00** (75.43)

Denotes statistically significant child/parent discrepancy at *** p < .001, ** p < .01, or * p < .05.

### Relationship between parent QOL and parent proxy-reported child QOL

For the total sample, there were statistically significant correlations between parents' ratings of their own QOL on the SF-36 measure and their ratings of the child's QOL on the PedsQL™ (0.32 to 0.37, Table 4). Within the three age groups, statistically significant correlations between parent QOL and parent proxy-rated child QOL were found for the middle age group (6.5 to 7.5 years).

**Table 4:** Correlations between parental QOL and parent-rated child QOL

	**Spearmans correlation coefficient (ρ)**			
**Scale**	**Total sample (n = 103)**	**5.5 – 6.5 years (n = 29)**	**6.5 – 7.5 years (n = 34)**	**7.5 – 8.5 years (n = 40)**
SF-36 Total Score to:				
PedsQL™ Total Score	0.37***	0.32	0.47**	0.30
PedsQL™ Physical Health	0.32***	0.34	0.36*	0.25
PedsQL™ Psychosocial Health	0.36***	0.24	0.49**	0.31

Denotes statistically significant correlation, at *** p < .001, ** p < .01, or * p < .05.

## Discussion

The results of this study show that agreement between child and parent proxy-reports of child QOL in healthy populations can be affected by the domains investigated, the age of children, and parents' own QOL.

Consistent with our predictions parent-child agreement on the PedsQL™ was low, with ICC values ranging from 0.02 to 0.23. Differences in levels of parent-child consistency were found when analyses were performed separately by age group and domain type. Statistically significant parent-child correlations were found for the oldest age group (7.5 to 8.5 years) for Total Score and Psychosocial Health, and for Physical Health scores in the youngest age group (5.5 to 6.5 years). This result is consistent with the findings of Eiser and Morse [16] who found that parent-child agreement can differ across domains investigated (i.e., higher agreement for physical aspects of health vs. emotional aspects). However our results showed domain and age differences in correlational consistency between child and parent ratings (i.e., higher agreement for younger age on physical health, compared to higher agreement for older age on psychosocial aspects of health).

We examined parent-child agreement on the PedsQL™ using median difference testing in addition to correlational consistency between child and parent ratings. Consistent with our expectations there were median differences between children's and parents scores. Parents reported better child QOL than did their children on the PedsQL™, and this finding is consistent with previous research with similar populations [e.g., [13,18,19]].

Parent-child median differences were largest for the older age groups, whereas parent-child scores were not different for the youngest age group (5.5 to 6.5 years). This result contradicts findings from other researchers who have shown agreement increasing with child age [e.g., [20,21]]. However we included healthy children and their parents, but previous researchers tested children with asthma [20] and children with epilepsy [21]. Eiser and Morse [16] found that parent-child agreement can be affected by children's illness status, therefore the difference in populations may account for the variation in results between studies.

We also considered the relationship between parents' own QOL on the SF-36 and their ratings of their child's QOL on the PedsQL™. Although correlations give no information about the causal direction of a relationship, we found statistically significant correlations between parents' own QOL ratings and their ratings of their child's QOL. These results are consistent with the findings of Eiser, Eiser, and Stride [22] and Goldbeck and Melches [23]. This correlation was only statistically significant for the middle age group (6.5 – 7.5 years). Future research needs to explore these subtle age differences in parent-child agreement in more detail.

Our results have implications for the measurement of child QOL and assessment of agreement between parents' and children's reports. We found parent-child agreement can be effected by the types of domains investigated and the ages of children in the sample. Our use of two different statistical methods allowed consideration of both the correlational consistency and the mean differences between parent proxy-report and child self-report scores to be considered. Our findings suggest that differences in the levels of parent-child agreement across previously reported studies may be either: 1) an artefact of statistical methods used; or 2) affected by the different ages of children in their sample populations.

There is a need for further research to explore whether parent-child agreement is dependent on additional factors such as relationship to child (i.e., mother vs. father), the mental health of the parent themselves, and for sick populations by different disease types (e.g., asthma vs. cancer vs. epilepsy). The optimal predictors of high or low parent-child agreement remain to be conclusively determined [26]. In addition, future researchers should provide details of both correlation consistency and means difference testing when investigating parent-child agreement. Using more than one statistical method can help provide meaningful data as high correlations between scores do not necessarily indicate high agreement between raters [26]. Correlations provide a criterion of relative agreement, but researchers also need an indicator of differences in group mean scores [19].

## Conclusion

The results from this study suggest that domains assessed and ages of children can effect parent-child agreement levels. In addition correlational consistency and mean differences in scores can provide different information on levels of agreement in ratings. Our findings support previous researchers [10,15,16,23] suggestions of future research to systematically examine the predictors of agreement levels between child and parent proxy-reported child QOL (such as child age or gender, relationship to child, health status, disease type).

## Competing interests

The author(s) declare that they have no competing interests.

## Authors' contributions

JC conceived the study, collected the data, conducted the analysis, drafted and revised the manuscript. CE and MB both contributed to the design of the study; interpretation of the data and analyses; and revised the article for important intellectual content. All authors gave final approval of the version to be published.
